# Association of hormone replacement therapy and the risk of knee osteoarthritis: A meta-analysis

**DOI:** 10.1097/MD.0000000000032466

**Published:** 2022-12-23

**Authors:** Wen-Yuan Hou, Cai-Yu Zhu, Yi-Fan Gu, Lei Zhu, Zheng-Xin Zhou

**Affiliations:** a Department of Orthopedics, The First Affiliated Hospital of Anhui University of Traditional Chinese Medicine, Hefei, China; b Department of Graduate School, Anhui University of Chinese Medicine, Hefei, China.

**Keywords:** association, hormone replacement therapy, HRT, knee OA, osteoarthritis

## Abstract

The relationship between hormone replacement therapy (HRT) and osteoarthritis is controversial in epidemiological studies. With the aim of better understanding the effect of HRT use, this first meta-analysis was implemented to explore the association of HRT and knee OA.

**Methods:**

A series of data is retrieved from Web of Science, PubMed, and Embase databases to observe the association of HRT and knee osteoarthritis up to December 2021. Two separated reviewers chose the research, extracted the data, and evaluated the study quality. Pooled estimates of 95% CI and HRs were acquired through a random-effects model.

**Results:**

Finally, there existed 13 pieces of research, containing one case-control research, four cross-sectional pieces of research, as well as eight cohort pieces of research, involving 2573,164 participants. The overall results showed that the use of HRT was related to a raised risk of knee OA (HR = 1.24, 95% CI 1.07–1.45). And the pooled analysis showed a statistically significant raised risk of knee joint replacement (HR = 1.30, 95% CI 1.09–1.54) when using HRT. In addition, the outcome exhibits the raised knee OA risk for the present users of HRT (HR = 1.40, 95% CI 1.16–1.68) according to HRT status. In the past users of HRT, the augment of knee OA risk was not statistically evident (HR = 1.16, 95% CI 0.94–1.42).

**Conclusion:**

We observed that HRT use was related to a raised knee OA risk. Furthermore, future studies might focus on relevant mechanistic to verify our observed associations.

## 1. Introduction

Osteoarthritis (OA) is one of the most prevalent arthritis. It involves joint inflammation and main architectural changes, resulting in functional damage and pain, especially in knee OA. Which affects approximately 260 million people around the world,^[1]^ with A high prevalence ranging from 6.8% to 30% across regions and countries.^[2]^ According to a recent study, it is reported that the incidence rate of symptomatic knee OA is 8.1% in Chinese patients over 45 years old. The incidence rate is increasing with the increase of age by using a nationwide survey.^[3]^ Furthermore, as much as half of the elderly ≥50 years old reported the pain of knee within a year, and one quarter possesses disabling and severe knee pain, which had a great impact on the ability to carry out daily activities,^[4,5]^ causing huge economic burden to person and society.

There exist numerous factors affecting the incidence of knee OA, for instance, the existence of hand former knee injury, raised BMI, intense physical activity, age, female gender, some physical and occupational activities (like squatting and kneeling) together with raised BMD.^[6–10]^ The meta-analysis of research based on predominantly Western populations presented the greatest difference in the incidence of OA between men and women, with a higher prevalence and higher disease severity in women compared to men in the knee joint, especially after menopause.^[11,12]^ These findings led researchers to suppose that hormonal factors may exert an important role in developing female knee osteoarthritis. Furthermore, several papers suggested that reproductive and hormonal factors might be responsible for the increased incidence of KOA in women. In a study of 224 Australian women, hormone replacement therapy (HRT) was observed to be a KOA risk factor.^[13]^ While some research showed that there was no relationship between HRT and knee OA.^[14,15]^ Thus, the relationship between HRT and knee OA was still unclear. In addition, up to this time, there was no evidence-based research on the influence of HRT use on knee OA. With the aim of better understanding the effect of employing HRT, this first meta-analysis was implemented to explore the association of HRT and knee OA.

## 2. Methods

### 2.1. Study registration

The study protocol was registered with the International Prospective Register of Systematic Reviews (PROSPERO) (number CRD42022334558).

### 2.2. Search strategy

Our meta-analysis adhered to the guidelines of PRISMA.^[16]^ We searched Web of Science, Embase, and PubMed databases up to December 2021 by using the terms “estrogen replacement therapy,” “hormone replacement therapy,” “hormone therapy,” “HRT” as well as “menopausal hormone therapy.” For each database, the detailed information of electronic search strategy is revealed in Table S1, Supplemental Digital Content, http://links.lww.com/MD/I215. All of the researches evaluating the association between knee OA and HRT use were qualified without language limitations. Search the list of references for each article and check the associated reviews manually for further determining underlying relevant research.

### 2.3. Study selection

We included all of the researches that conformed to the below criteria: previously healthy people diagnosed with knee OA compared with people without knee OA; patients confirmed radiographically or by accepting knee replacement; the exposure was HRT; study design was cross-sectional or cohort or case-control researches; 95% CIs (or information to count them) and risk estimates were reported; where there was a possible overlap between two or more studies, only the one with more detail was included; and case reports, abstract papers only, repetitions, reviews, animal studies, and case series are excluded.

### 2.4. Data extraction

The below data was extracted by two independent investigators (W.-Y.H. and C.-Y.Z.) from eligible articles, including country, year of publication, author, sample size, age, research design, the definition of knee OA, HRT type, exposure category adjustment factors, HR estimate, and 95% CI. If the research afforded to exceed one risk estimate, we extracted a risk estimate modulating for the maximum confounding factors number. Furthermore, the present users refer to those who utilize HRT at the baseline. Past users refer to those who have reported the use of HRT at any time before but have not reported it at the baseline.

### 2.5. Study quality assessment

The quality evaluation of cross-sectional researches and case-control were implemented with the revised version of the Newcastle–Ottawa scale (NOS) for the case-control researches.^[17]^ The NOS for the cohort research was conducted to assess cohort study by two investigators (W.-Y.H. and C.-Y.Z.). We believe that researches with 6 points or more are of high quality. Differences are resolved through a third investigator or discussion.

### 2.6. Statistical methods

Since the results are rare relatively, RRs and ORs were regarded as approximate values of hazard ratios (HRs). Pooled estimates of 95% CI and HRs were acquired through a random-effects model, in which limited maximum likelihood estimates were employed for assessing inter-study heterogeneity.^[18,19]^ Furthermore, the subgroup analysis was implemented stratified by research design, country, statistical type, diagnosed method, and current or past users. For evaluating the stability of the outcomes, a sensitivity analysis was performed via excluding a study at one time. Egger’s test and funnel plot were utilized to estimate the publication bias. Stata 16.0 software was employed for the statistical analysis. *P* < .05 was considered as statistical significance.

## 3. Results

### 3.1. Characteristics of the included studies

**Figure 1.**
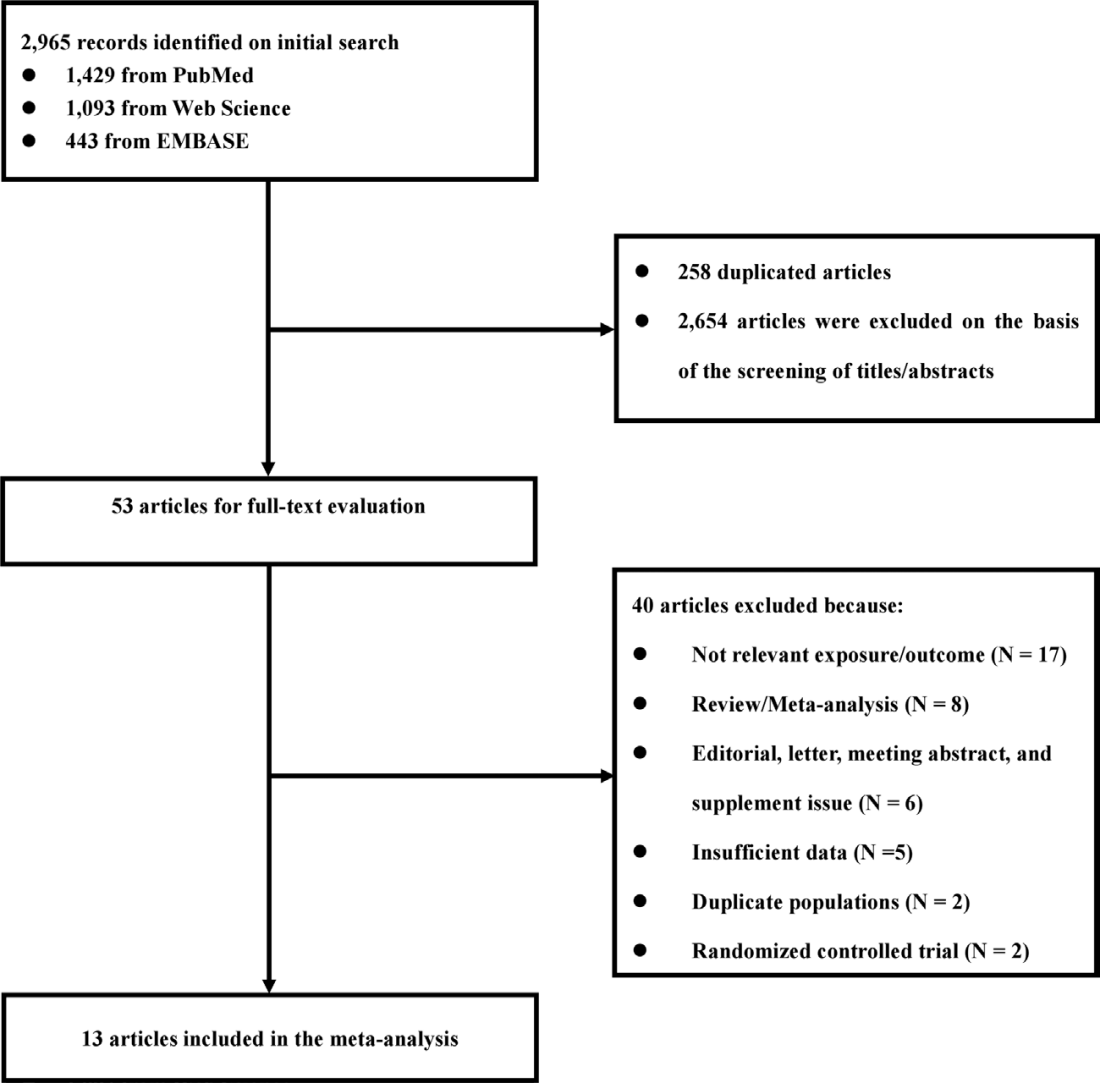
The flow chart of selection of included studies.

The research selection and flow chart of literature retrieval are reflected in Figure 1. 2965 titles and abstracts were determined and evaluated, eventually, there existed 13 studies^[13,20–31]^ containing one case-control research, four cross-sectional research, and eight cohort research, involving 2573,164 participants, meeting the included criteria. There were 6 studies describing the current use and past use respectively. Furthermore, there were four studies from Asia, three studies from Europe, two pieces of research from Australia, and two studies from the US. In addition, 6 studies were reporting ORs, four studies reporting HRs, and three studies reporting RRs (Table 1).

**Table 1:** Characteristics of the studies investigating the association between hormone replacement therapy and knee osteoarthritis.

Author, year, country [reference]	Study design	Age	Participants/cases	KOA definition	HRT type	Exposure category	Adjustments
Eun Y, 2021, Korea	Cohort study	61.4 ± 8.3 (mean ± SD)	1134,680/60,670	Knee replacement	HRT	Never use: HR 1	Age, reproductive span, parity, duration of breastfeeding, duration of OC use, alcohol consumption, smoking, regular exercise, income, BMI, hypertension, diabetes mellitus, and dyslipidemia
						<2 yr: HR 1.06 (1.03–1.09)	
						2–5 yr: HR 1.12 (1.07–1.17)	
						5+ yr: HR 1.22 (1.16–1.28)	
Leung YY, 2019, China	Cohort study	45–74 (range)	63,257/1645	Knee replacement	HRT	Never use: HR 1	Age at interview, dialect, year of interview, educational level, smoking status, BMI, total PA duration, sleep duration and sitting duration, baseline history of self-reported, diabetes, hypertension, coronary artery disease, and stroke, number of children, age at menarche, and age at menopause
						Ever use: HR 1.08 (0.87–1.36)	
Jung JH, 2018, Korea	Cross-sectional study	≥50 (range)	4766/1153	Radiographic KOA	HRT	Use: OR 0.70 (0.50–0.99)	MHT duration, age, obesity, menarche and menopause age, HTN, DM, alcohol intake, smoking status, and socioeconomic status
Zhou M, 2018, China	Cross-sectional study	62.6 ± 8.6 (mean ± SD)	7510/640	Radiographic KOA	HRT	History of use: OR 1.65 (1.11–2.47)	Age, BMI, education, alcohol drinking, physical exercise, work posture, and shift work
Hussain SM, 2018, Australia	Cohort study	Cases: 57.6 ± 7.3 (mean ± SD) Controls: 54.4 ± 8.6 (mean ± SD)	22,289/1208	Knee replacement	HRT	Never use: HR 1	BMI at midlife, change in BMI (early to midlife), country of birth, education level, vigorous PA, and smoking status
						Past use: HR 0.99 (0.79–1.25)	
						Current use: HR 1.37 (1.14–1.64)	
						<1 yr: HR 1.12 (0.91–1.39)	
						≥1 yr: HR 1.30 (0.99–1.72)	
Hellevik AI, 2017, Norway	Cohort study	55.7 (mean)	30,289/430	Knee replacement	HRT	Never use: HR 1	Age, BMI, smoking, PA, parity, menopausal status
						Past use: HR 1.42 (1.06–1.90)	
						Current use: HR 1.25 (0.90–1.73)	
						Local use: HR 1.23 (0.90–1.68)	
						Systemic use: HR 1.40 (1.03–1.90)	
						Years of use: HR 1.03 (1.00–1.06)	
Liu B, 2009, UK	Cohort study	56 (mean)	1306,081/9977	Knee replacement	E, E + P, T	Never use: RR 1	Age, recruitment region, height, BMI, alcohol, smoking, socioeconomic status, age at menarche, oral contraceptive use, parity, hysterectomy and bilateral oophorectomy
						Past use: RR 1.39 (1.29–1.49)	
						Current use: RR 1.58 (1.48–1.69)	
						<5 yr: RR 1.52 (1.37–1.68)	
						5–9 yr: RR 1.52 (1.39–1.66)	
						10+ yr: RR 1.72 (1.56–1.89)	
						E: RR 1.48 (1.35–1.63)	
						E + P: RR 1.68 (1.54–1.83)	
						T: RR 1.44 (1.20–1.74)	
Szoeke CEI, 2006, Australia	Cohort study	49.66 ± 2.47 (mean ± SD)	224/49	Radiographic KOA	HRT	Use: RR 1	Age, BMI, PA at ages 20–29, smoking status
						Never use: RR 2.90 (0.80–11.60)	
Von Mühlen D, 2002, USA	Cross-sectional study	72 (mean)	1001/224	The American College of Rheumatology (ACR) standard criteria	E	0–<1 yr: OR 1	Age, smoking, exercise, BMI, and type of menopause
						≥1 yr: OR 1.30 (0.93–1.81)	
Hart DJ, 1999, UK	Cohort study	54.1 ± 5.9 (mean ± SD)	715/95 for knee osteophytes; 644/81 for knee JSN	Radiographic KOA	E	Keen osteophytes Ever use: OR 0.73 (0.32–1.67) Current use: OR 0.41 (0.12–1.42)	Hysterectomy, smoking, PA, knee pain, and social class
						Knee JSN Ever use: OR 1.17 (0.59–2.33) Current use: OR 1.88 (0.86–4.11)	
Sandmark H, 1999, Sweden	Case-control study	55–70 (range)	584/300	Knee replacement	E	Use: RR 1.8 (1.2–2.6)	Age, physical load, body weight, and sports
Zhang Y, 1998, USA	Cohort study	63–91 (range)	551/71	Radiographic KOA	E	Never use: OR 1	Age, BMI, femoral neck bone mineral density, PA Index, weight change, knee injury history, smoking status, and baseline Kellgren and Lawrence grade
						Past use: OR 0.8 (0.5–1.4)	
						Current use: OR 0.4 (0.1–3.0)	
Sowers MF, USA, 1996	Cross-sectional study	24–45 (range)	573/21	The Kellgren and Lawrence grading system	HRT	Use: OR 2.56 (0.68, 9.5)	Age, weight, height, BMI, BMD, menopausal status, activity level, smoking, blood pressure status, and alcohol consumption

E = estrogen, HR = hazard ratio, HRT = hormone replacement therapy, KOA = knee osteoarthritis, OR = odds ratio, P = progesterone, RR = risk ratio, T = tibolone.

For all of the included researches, the methodological quality is displayed in Tables 2 and 3. In accordance with the NOS outcomes, there were one, seven, three, two studies having six, seven, eight, and nine stars respectively. Based on the modified NOS outcomes, there were one studies having six stars, one studies having seven stars, two studies having eight stars, and one studies having nine stars respectively.

**Table 2:** Quality of included cohort studies based on the Newcastle–Ottawa scale.

Study	Selection				Comparability	Outcome			Total score
	Representativeness of exposed cohort☆	Selection of non-exposed cohort☆	Exposure ascertainment☆	Demonstration that outcome of interest was not present at start of study☆	Comparability of cases and controls on basis of design or analysis☆☆	Outcome assessment☆	Adequate follow-up☆	Loss to follow-up rate☆	
Eun Y, 2021, Korea	☆	☆	☆	☆	☆☆	☆	–	–	7
Leung YY, 2019, China	☆	☆	☆	☆	☆☆	☆	☆	☆	9
Hussain SM, 2018, Australia	☆	☆	☆	☆	☆☆	☆	☆	–	8
Hellevik AI, 2017, Norway	☆	☆	☆	☆	☆☆	☆	–	–	7
Liu B, 2009, UK	☆	☆	☆	☆	☆☆	☆	–	–	7
Szoeke CEI, 2006, Australia	–	–	☆	☆	☆☆	☆	☆	☆	7
Hart DJ, 1999, UK	☆	☆	☆	☆	☆	☆	–	☆	7
Zhang Y, 1998, USA	☆	☆	☆	☆	☆☆	☆	–	–	7

**Table 3:** Quality of included case-control studies and cross-sectional studies based on the modified version of Newcastle–Ottawa scale.

Study	Selection				Comparability	Outcome			Total Score
	Adequate case definition☆	Representativeness of cases☆	Selection of controls☆	Definition of controls☆	Comparability of cases and controls on basis of design or analysis☆☆	Exposure ascertainment☆	Same method of ascertainment for cases and controls☆	Non-response rate☆	
Jung JH, 2018, Korea	☆	–	–	☆	☆☆	☆	☆	☆	7
Zhou M, 2018, China	☆	–	–	☆	☆☆	☆	☆	–	6
Von Mühlen D, 2002, USA	☆	☆	☆	☆	☆☆	☆	☆	☆	9
Sandmark H, 1999, Sweden	☆	☆	☆	☆	☆☆	☆	☆	–	8
Sowers MF, 1996, USA	☆	–	☆	☆	☆☆	☆	☆	☆	8

### 3.2. Association of HRT and knee OA

**Figure 2.**
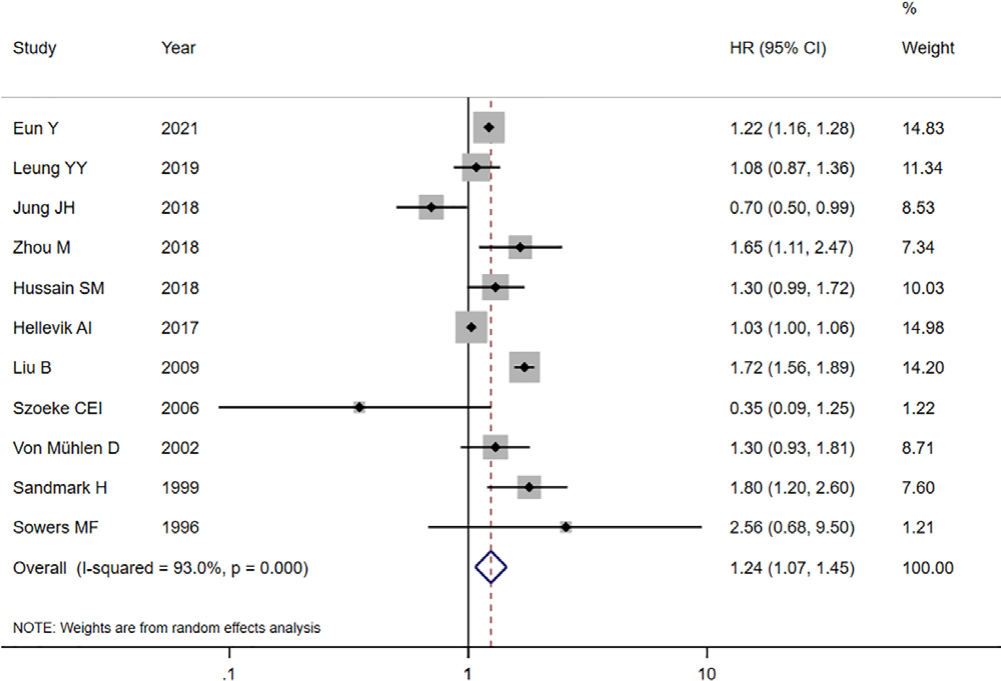
The pooled results of the relationship between HRT use and risk of knee OA. CI = confidence interval, HR = hazard ratio, HRT = hormone replacement therapy, OA = osteoarthritis.

The pooled results showed that HRT use was related to a raised knee OA risk (HR = 1.24, 95% CI 1.07–1.45, *P* < .05) (Fig. 2). There were six studies describing that participants accepted knee joint replacement, Figure S1, Supplemental Digital Content, http://links.lww.com/MD/I216 showed a statistically significant raised risk of the knee joint replacement (HR = 1.30, 95% CI 1.09–1.54) when using HRT. The pooled analysis exhibits the raised knee OA risk for the present users of HRT (HR = 1.40, 95% CI 1.16–1.68) according to HRT status. In the past users of HRT, the increase of knee OA risk was not evident statistically (HR = 1.16, 95% CI 0.94–1.42) (Figure S2, Supplemental Digital Content, http://links.lww.com/MD/I217). When the results were stratified by study design, a statistically evident raised knee OA risk was found only in cohort studies (HR = 1.22, 95% CI 1.02–1.47, *P* < .05), although the increased risk was not evident statistically in the cross-sectional researches, the HR point estimates were similar (Figure S3, Supplemental Digital Content, http://links.lww.com/MD/I218). In accordance with the subgroup analysis stratified through various regions. The HRs and 95% CIs were 1.11 (0.88–1.41), 0.79 (0.23–2.76), 1.45 (0.94–2.21), 1.35 (0.98–1.87) for studies’ participants from Asia, Australia, Europe and USA, respectively (Figure S4, Supplemental Digital Content, http://links.lww.com/MD/I219).

Furthermore, the pooled analysis showed statistical significance on the raised knee OA risk when participants used HRT by using HRs (HR = 1.14, 95% CI 1.00–1.30, *P* < .05). While it showed no obvious relationship between HRT use and the risk of knee OA by utilizing ORs (HR = 1.55, 95% CI 1.05–2.29) or RRs (HR = 1.23, 95% CI 0.77–1.97).

### 3.3. Sensitivity analysis

Sensitivity analysis exhibited that the combined HRs were not markedly affected by any single research in all the results, suggesting that our outcomes were robust statistically (Figure S5, Supplemental Digital Content, http://links.lww.com/MD/I220].

### 3.4. Publication bias

**Figure 3.**
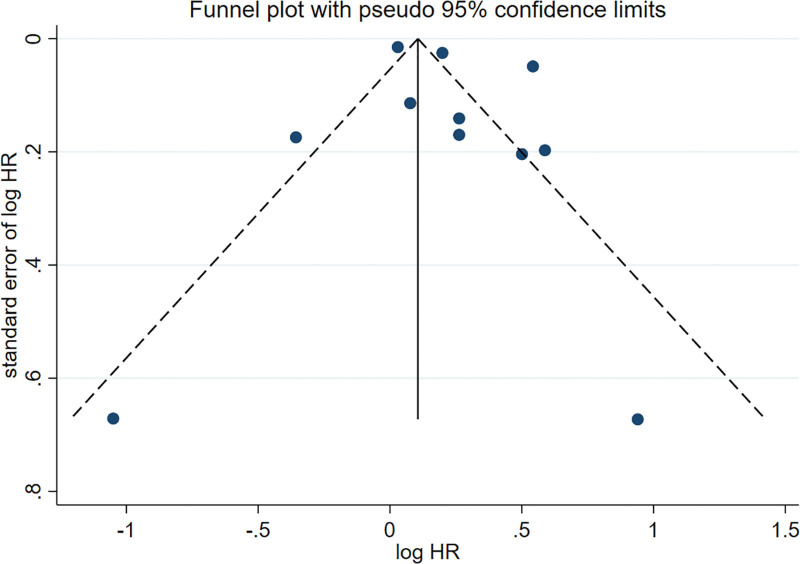
The funnel plot of the meta-analysis of included studies. HR = hazard ratio.

Publication bias was evaluated by utilizing a funnel plot. There is no obvious publication bias (Fig. 3). The statistical analysis indicated no evident publication bias (Egger’s test *P* = .31).

## 4. Discussion

Epidemiological studies have found that reproductive history and hormonal factors concerning osteoarthritis, are contradictory.^[12,20,31]^ As a result, this first meta-analysis was implemented for assessing the association between risk of knee OA and HRT, which has not been researched before. We explored the influence of HRT use on the knee OA risk involving 2573,164 participants. Combining the outcomes of 13 research, it can be observed that the use of HRT was related to a raised knee OA risk. Furthermore, finding from our research showed that users of systemic HRT had higher risk of knee replacement compared to never users. Which is in accordance with the results by Liu et al^[23]^ As a report, the utilization of postmenopausal hormones was related to a raised incidence of hip and knee replacements and an increased incidence of knee replacements. A multi-center study reported that an increase in the number of pregnancies was positively related to an increased incidence of KOA.^[14]^ This indicated that altered sex hormones may affect bone conversion through estrogen receptors in the musculoskeletal system and disrupt the metabolic balance of bone, as well as cartilage. Previous studies presented significant associations between reproductive factors, OCP use, total knee arthroplasty risk in normal-weight but non-obese or overweight women. The mechanism is probably that reproductive factors are related to endothelial dysfunction, inflammation and obesity.^[32,33]^ The exact mechanism by which HRT increases the risk of knee osteoarthritis is not yet clear and needs to be further explored.

The results of subgroup analysis stratified by past and current use showed the increased risk of knee OA for current HRT users. A recent study performed by Hellevik et al^[22]^ also found that the present systemic HRT users were at higher risk of total knee replacement compared to never users. In the past users of HRT, the increase of knee OA risk was not evident statistically. Besides, a former study exhibited that people who currently utilize HRT were older than those in the past (53.9 ± 7.0 years and 50.3 ± 7.5 years), owing to in the developed countries, the average age at menopause was 51 years. These data indicated that past users of HRT began to utilize HRT in the process of the menopausal transition, whereas current users of HRT started using it approximately 3 years after menopause.^[22]^ This may be owing to the onset of HRT in late menopause, which is related to a raised knee OA risk.

Moreover, the subgroup analysis stratified by study design presented a statistically evident raised knee OA risk, found only in cohort studies, not in case-control and cross-sectional research. Since there existed only one case-control study and four cross-sectional studies, the sample size is very small, which might have an impact on the outcome. Alternatively, there was no significant difference in the relationship between the HRT users from different regions or countries and the knee OA risk.

The advantages of this meta-analysis include its large sample size, furthermore, we conducted a careful subgroup analysis stratified by the country, the current use or past use, the study design, the statistical type, and definition of knee OA, which have not been done before. However, there were some limitations in our study. To be first, the included researches differed in the approaches of evaluating HRT and the subsequent timing differed. Second, despite numerous studies having modulated for significant risk factors, residual confounding factors associated with HRT use might affect the individual studies outcomes. Furthermore, some studies cannot exclude the possibility that recall bias might have impacts on some of the covariates, such as age at menarche. Hence, it still needs more research on it to discover the correlation between knee osteoarthritis and hormone replacement therapy.

## 5. Conclusion

The research observed that HRT use was related to a raised knee OA risk. Besides, our outcomes revealed that present HRT users have a raised knee OA risk. Additionally, future research should focus on the mechanisms by which HRT affects knee OA.

## Acknowledgments

We would like to express our gratitude to our all team members for their assistance and constant support provided by them.

## Author contributions

**Critical revision of the article:** Yi-Fan Gu, Zheng-Xin Zhou.

**Data analysis and interpretation:** Wen-Yuan Hou, Lei Zhu, Cai-Yu Zhu.

**Data collection:** Wen-Yuan Hou, Cai-Yu Zhu.

**Final approval of the article:** All authors.

**Paper writing:** Wen-Yuan Hou.

**Study concept and design:** Wen-Yuan Hou, Zheng-Xin Zhou.

## Supplementary Material
