# Supplementary material for: Association of hormone replacement therapy and the risk of knee osteoarthritis: A meta-analysis
Source: Medicine (Baltimore). 2022 Dec 23;101(51):e32466. doi: 10.1097/MD.0000000000032466 (PMC9794300; doi:10.1097/MD.0000000000032466)
Supplement: Supplementary file 1 [file medi-101-e32466-s001.pdf]

Supplementary table S1. Search strategy used in the meta-analysis

**Literature search strategy in PubMed**
#1 hormone replacement therapy OR hormone therapy OR estrogen replacement therapy OR menopausal hormone therapy OR HRT<br>#2 knee osteoarthritides OR knee osteoarthritis OR knee arthritis<br>#3 #1 AND #2
**Literature search strategy in EMBASE**
#1 hormone replacement therapy OR hormone therapy OR estrogen replacement therapy OR menopausal hormone therapy OR HRT<br>#2 knee osteoarthritides OR knee osteoarthritis OR knee arthritis<br>#3 #1 AND #2
**Literature search strategy in Web of Science**
#1 Search TS= (hormone replacement therapy OR hormone therapy OR estrogen replacement therapy OR menopausal hormone therapy OR HRT)<br>#2 Search TS= (knee osteoarthritides OR knee osteoarthritis OR knee arthritis)<br>#3 #1 AND #2
