# Supplementary figures and images for: Association of hormone replacement therapy and the risk of knee osteoarthritis: A meta-analysis
Source: Medicine (Baltimore). 2022 Dec 23;101(51):e32466. doi: 10.1097/MD.0000000000032466 (PMC9794300; doi:10.1097/MD.0000000000032466)

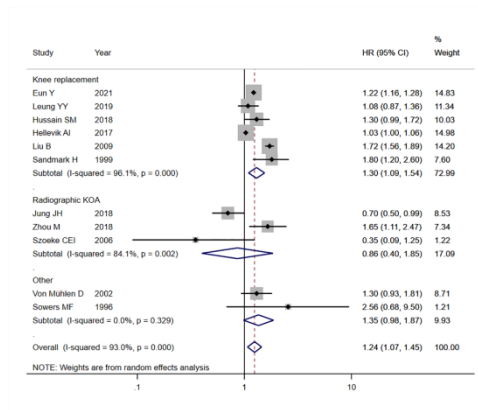

Supplement Figure 1: The subgroup analysis results stratified by knee OA definition.

Supplement: Supplementary file 2 [file medi-101-e32466-s002.pdf]

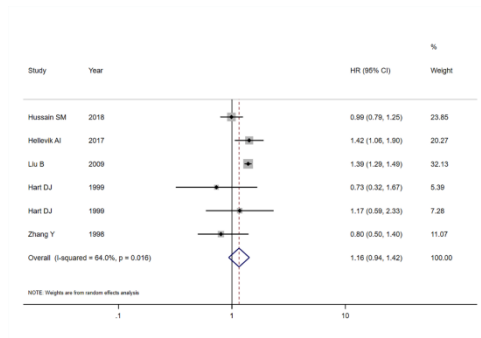

Supplement Figure 2: The subgroup analysis results stratified by use condition.

Supplement: Supplementary file 3 [file medi-101-e32466-s003.pdf]

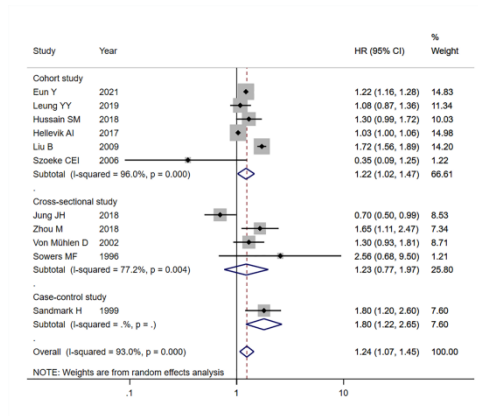

Supplement Figure 3: The subgroup analysis results stratified by study design.

Supplement: Supplementary file 4 [file medi-101-e32466-s004.pdf]

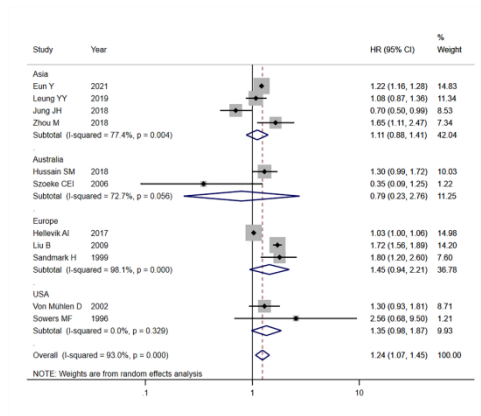

Supplement Figure 4: The subgroup analysis results stratified by country.

Supplement: Supplementary file 5 [file medi-101-e32466-s005.pdf]

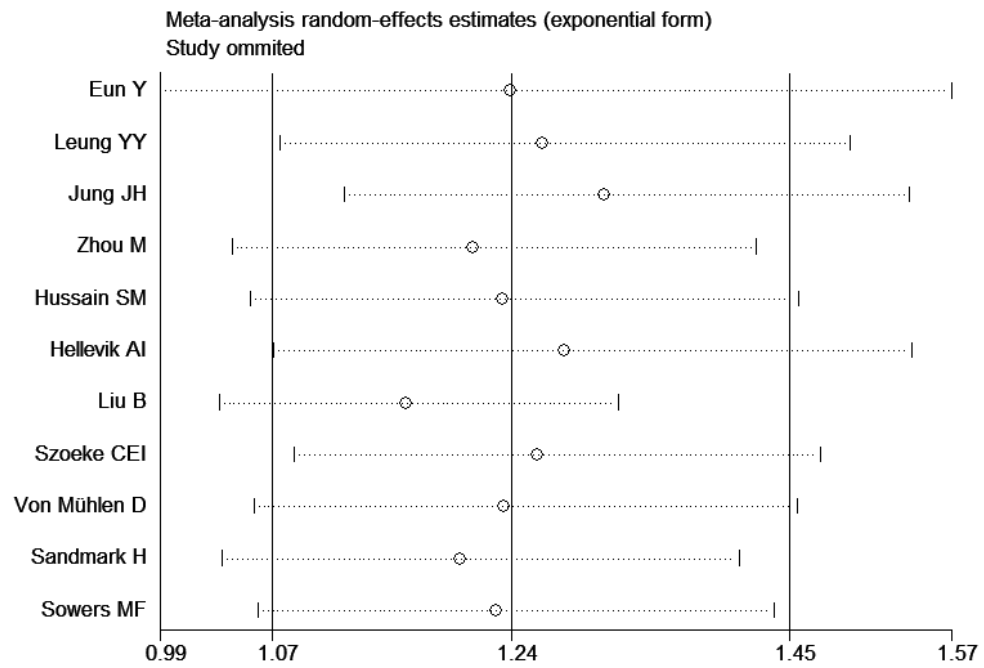

Supplement Figure 5: The sensitivity analysis of the meta-analysis of included studies.

Supplement: Supplementary file 6 [file medi-101-e32466-s006.pdf]
